# Emotional intelligence mediates the relationship between sport type and social anxiety in adolescents

**DOI:** 10.3389/fspor.2026.1868592

**Published:** 2026-06-05

**Authors:** Helder Miguel Fernandes, Henrique Costa, Pedro Tiago Esteves, Teresa Fonseca, Samuel Honório, Aristides Miguel Machado-Rodrigues

**Affiliations:** 1 Polytechnic Institute of Guarda, Guarda, Portugal; 2 Sport Physical Activity and Health Research & Innovation CenTer (SPRINT), Guarda, Portugal; 3 Polytechnic Institute of Castelo Branco, Castelo Branco, Portugal; 4 Faculty of Sport Sciences and Physical Education, University of Coimbra, Coimbra, Portugal; 5 Interdisciplinary Centre for the Study of Human Performance (CIPER), University of Coimbra, Coimbra, Portugal; 6 Research Centre for Anthropology and Health, University of Coimbra, Coimbra, Portugal

**Keywords:** adolescents, emotional intelligence, moderated mediation, social anxiety, sport participation

## Abstract

**Background:**

Adolescence is a developmental period marked by heightened social anxiety vulnerability. Organized sport may protect mental health, but the psychological mechanisms linking sport type to social anxiety remain unclear.

**Objective:**

This study examined whether specific emotional intelligence dimensions mediate the association between sport type and social anxiety among adolescents, and whether sex or age moderates these relationships.

**Methods:**

The sample comprised 1,036 adolescents (603 girls and 433 boys), aged 12–17 years. Participants completed self-report measures assessing sport participation (no sport = 684; individual = 156; and team = 196), emotional intelligence, and social anxiety. Mediation and moderated-mediation analyses were conducted using bootstrapped regression models.

**Results:**

The use of emotion emerged as the most consistent mediator between sport participation and lower levels of social anxiety. Both individual and team sports were indirectly associated with lower fear of negative evaluation, reduced social avoidance and distress through a greater use of emotion. Team sport participation also showed a direct positive association with fear of negative evaluation, indicating inconsistent mediation. Sex showed no significant moderation effect. Age influenced certain direct and indirect pathways, with team sport participation showing a transient positive association with fear of negative evaluation during early adolescence, buffered by stable protective indirect effects of use of emotion across ages.

**Conclusions:**

These findings indicate that sport participation types were indirectly associated with lower social anxiety in adolescents through adaptive use of emotion, while also suggesting that team sport contexts may amplify evaluative pressures that increase fear of negative evaluation during early adolescence.

## 1 Introduction

Adolescence is a critical developmental period of life characterized by significant physiological, social, and psychological changes that often lead to increased levels of internalizing symptoms (1–3). As the transition from childhood to adulthood spans a greater portion of the lifespan, understanding its key influencing factors and associated challenges becomes increasingly important. Among these challenges, social anxiety, defined by an intense and persistent fear of negative evaluation, humiliation, or embarrassment within social or performance-related situations, is particularly prevalent (3, 4). Indeed, social anxiety disorder is one of the most common mental disorders among youth, with an estimated global prevalence of 8.3% (5), and its onset is typically observed during early adolescence (2, 6). The most common symptoms of social anxiety are not merely transitory but exhibit cross-temporal stability and are substantially and consistently related to an increased risk for subsequent depression and psychosocial impairment (7, 8). Considering that adolescents are particularly sensitive to social approval and peer interactions, identifying protective factors and the psychological mechanisms that may help alleviate social distress is a crucial public health priority (2, 9).

Organized sport has long been recognized as a significant social phenomenon and a distinct microcosm that facilitates developmental social interactions (10, 11). Participation in sport provides adolescents with structured opportunities to enhance self-confidence, engage in peer relationships, and manage stress in a community context (10, 12). Systematic reviews and meta-analyses have shown that symptoms of anxiety and depression are significantly lower among adolescents who participate in organized sports compared to those who do not (13, 14). However, the magnitude of this association is often small and may depend on the context and quality of the involvement (13, 15). For example, the specific type of sport participation—traditionally categorized as individual or team-based—may elicit distinct psychological outcomes due to differences in social structures and interactions within and across training and competition settings (15–17).

Theoretical frameworks suggest that team sports, characterized by interdependent cooperation and shared collective goals, may provide a crucial buffer against fears of social evaluation and internalizing symptoms (17–19). Available research (15, 17) has shown that athletes participating in team sports often report lower levels of social avoidance and distress compared to their counterparts in individual sports. In contrast, participation in individual sports may be associated with higher levels of perceived social avoidance and fear of negative evaluation, potentially due to personal accountability for competitive results and training-related pressures (17, 20, 21). Unsurprisingly, individual sports participants tend to prioritize autonomy and self-reliance, primarily strengthening psychological resilience through the development of self-efficacy rather than through collective mechanisms, such as social support (19). Despite the aforementioned contrasts, some cross-sectional studies have reported no significant differences in social anxiety between team and individual sport participants (21, 22), suggesting that regular participation may be the primary driver of social and mental health benefits, regardless of the sport type. Given these inconsistencies and limited evidence, there is a need to adopt a mediator-based approach to better understand how specific social and structural demands of different sports (team or individual) influence social anxiety among adolescents.

Drawing on recent evidence (19, 23, 24), emotional intelligence (EI) has emerged as a potential mediator between sport type and social anxiety in adolescents. Originally defined as the capacity to perceive, understand, and effectively regulate emotions to achieve personal and social goals, EI allows individuals to manage affective information adaptively (25). In the context of sports, higher levels of EI have been shown to have a small, yet significant, positive association with competitive performance, as well as helping athletes cope with competition-related stress (26, 27). Available evidence indicates that team-sport environments are particularly beneficial for the development of social-emotional skills (28, 29). Indeed, engaging in team sports usually requires athletes to coordinate their actions, negotiate conflicts, and use interpersonal skills, often resulting in higher expression of dimensions such as self-awareness and self-regulation compared to their individual-sport counterparts (19, 30). Subsequently, the theoretical association between EI and social anxiety is well-documented, with studies consistently demonstrating a negative correlation between the two dimensions (31, 32). Recent studies indicate that EI may serve as a key mediator in reducing social anxiety by mitigating the intensity of negative emotions and improving interpersonal skills (32, 33). Adolescents with high EI are more able to interpret training stressors in an adaptive manner and are less likely to engage in maladaptive strategies, such as expressive suppression and increased rumination (9, 34). Additionally, research indicates that physical exercise can indirectly reduce social anxiety by improving emotional regulation and sports self-efficacy (9) or enhancing self-control and mental toughness among adolescents (35). Therefore, it is plausible to hypothesize that the specific demands of different types of sport (namely, team or individual sports) may promote higher EI, which in turn may serve as a psychological buffer against social anxiety symptoms. However, to the best of our knowledge, this mediation model has not yet been empirically tested.

The efficacy and relevance of this mediation model are likely conditional upon individual demographic factors, specifically sex and chronological age. Sex serves as a primary moderator in the development of both EI and social anxiety during adolescence (36, 37), with girls typically reporting higher levels of ability-EI, social anxiety, and fear of negative evaluation than boys. However, other studies suggest that male participants, particularly in certain individual or performance-focused contexts, may report comparable or even higher levels of fear of negative evaluation than their female counterparts (21, 38). Furthermore, research indicates that EI tends to increase with age during adolescence, driven by the expansion of social experiences and cognitive maturation (37, 39). However, studies also suggest that changes in specific dimensions of EI may follow nonlinear trajectories, with certain aspects, such as perceived adaptability or intrapersonal skills, potentially declining during late adolescence (40). Additionally, the impact of sports participation on social-emotional skills appears to change during adolescence. For younger adolescents, participation in sports is more associated with collaboration and task performance, while for older adolescents, it is more closely associated with emotional regulation (41). Notably, the specific relationship between sports participation and adolescents' social anxiety symptoms does not appear to be moderated by age or sex (42).

### 1.1 The present study

While prior research has explored the relationships among sports participation, EI, and/or symptoms of social anxiety, the specific mediating role of the different EI dimensions in the relationship between sport involvement and social anxiety has yet to be empirically tested, particularly during adolescence. In addition, participation in different types of sports (e.g., team or individual) has been distinctly associated with social and psychological outcomes due to fundamental differences in social structure and interaction dynamics, performance pressures, and personal accountability. Hence, further research is needed to clarify the complex relationships between sport type, EI, and social anxiety among adolescents.

**Figure 1:**
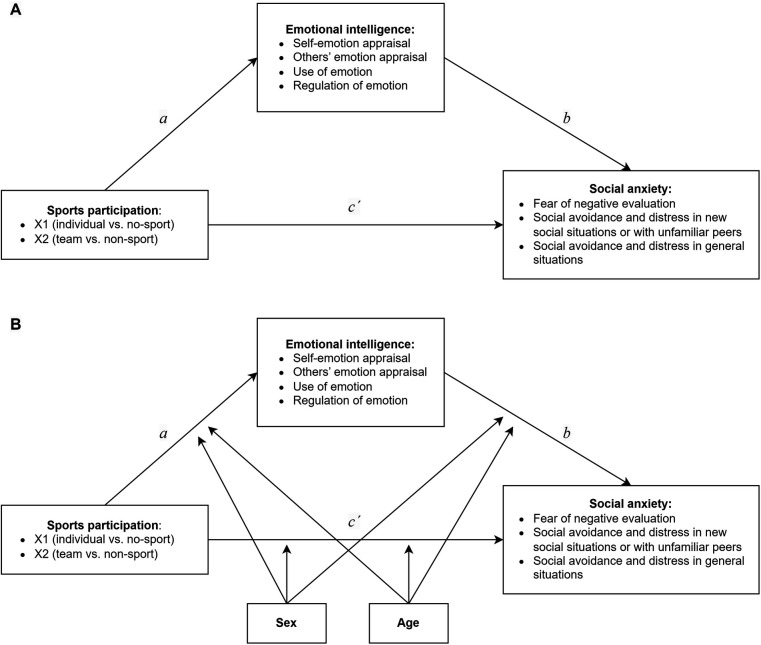
Mediation (panel **A**) and moderated-mediation model (panel **B**) of the conditional relationships of sports participation, emotional intelligence, and social anxiety.

Therefore, the aim of this study is twofold. First, we examine the extent to which various dimensions of EI (self-emotional appraisal, others' emotional appraisal, regulation of emotion, and use of emotion to facilitate performance) can mediate the association between sports participation and components of social anxiety in adolescents (including fear of negative evaluation, social avoidance and distress in new social situations or with unfamiliar peers, as well in general situations). Figure 1, panel A, illustrates the proposed mediation model. Second, we explore the potential moderating effects of adolescents' sex or age on the direct associations between sport type and the dimensions of social anxiety (paths *c*´), as well as on the indirect relationships mediated by the four dimensions of EI (paths *ab*). Figure 1, panel B, presents this proposed moderated-mediation model.

## 2 Materials and methods

### 2.1 Participants

This study included 1,036 adolescents (603 girls and 433 boys) with ages between 12 and 17 years (*M* = 14.34, SD = 1.49). All participants were enrolled in grades 7th through 12th of public schools in Portugal. About one-third of the sample (*n* = 352, 34.0%) reported participating in organized sports activities; among those, 196 adolescents (18.9% of the total sample) were involved in team sports, while 156 (15.1%) participated in individual sports. The inclusion criteria required participants to be aged between 12 and 17 years old, to be enrolled in one of the selected school classes, and to have documented informed consent from a parent or legal guardian. The age range of 12 to 17 years was selected because it aligns with the school-based sampling framework and allows for a focus on adolescents who are still legally considered minors in Portugal. This selection ensures greater developmental and legal uniformity while avoiding the inclusion of participants who have reached adulthood at age 18. Although a formal *a priori* power analysis was not conducted, the study was designed to recruit a large sample (*n* > 1,000) to enhance the precision and stability of parameter estimation in conditional process models, given the sample-size sensitivity inherent in mediation and moderated-mediation analyses (43–45).

This research project received ethical approval before data collection (Study Registration No. 0395700001/MIME).

### 2.2 Measures

Initially, participants were asked to provide self-report information regarding their sex, chronological age, and involvement in sports. Participation in organized sports was defined as activities conducted under the supervision of certified coaches within sports clubs. Sport participation was classified as individual sports (e.g., gymnastics, swimming, track and field, martial arts) or team sports (e.g., soccer, basketball, futsal, handball, volleyball).

Next, participants completed a series of Portuguese-validated self-report questionnaires to assess EI and social anxiety.

EI was measured using Wong and Law's scale (46). This 16-item instrument evaluates four dimensions: self-emotion appraisal (SEA, 4 items), others' emotion appraisal (OEA, 4 items), use of emotion (UOE, 4 items), and regulation of emotion (ROE, 4 items). Participants rated items on a 5-point Likert scale (1 = strongly disagree to 5 = strongly agree), with dimension scores ranging from 5 to 20. Higher scores reflect greater levels of EI. Omega reliability coefficients indicated acceptable internal consistency across all dimensions (*ω* = 0.73–0.80).

The Social Anxiety Scale for Adolescents (47) was used to assess participants' social anxiety symptoms. This instrument consists of 22 items, with 18 items specifically addressing social anxiety and 4 serving as filler (neutral) items. Responses were rated on a 5-point Likert scale, ranging from 1 (not at all) to 5 (all the time). This instrument includes three subscales: Fear of Negative Evaluation (FNE, 8 items, range between 8 and 40), Social Avoidance and Distress in New Situations (SAD-New, 6 items, range between 6 and 30), and Social Avoidance and Distress in General (SAD-Gen, 4 items, range between 4 and 20). Higher scores indicate greater symptoms of social anxiety. Internal consistency analysis revealed high omega reliability, ranging from 0.82 to 0.88.

### 2.3 Procedures

Authorization to assess adolescents was obtained from the ethics committee of the General Directorate of Education (Ministry of Education, Study Registration N° 0395700001/MIME), and parental signed consent was obtained for all participants. A multistage sampling procedure was employed to select the study sample. Public schools in the Portuguese midlands were purposively chosen, and after obtaining approval from the school directors, one to two classes from grades 7 to 12 were randomly selected from each school using a lottery system. Questionnaires were administered individually in quiet classroom settings. Participation was voluntary, with data collected anonymously and treated as confidential.

### 2.4 Data analysis

Descriptive statistics were computed, including means (M), standard deviations (SD), 95% confidence intervals (CI), as well as frequencies and percentages (%). Additionally, point bi-serial or Pearson correlations were calculated between the variables under analysis. The internal consistency of the scales was assessed using McDonald's omega coefficient (*ω*), with values of 0.70 or higher considered acceptable.

Mediation analyses were conducted using Model 4 in the PROCESS macro (version 5.0) for SPSS (43), with three separate analyses examining each social anxiety dimension as the outcome. These analyses employed the type of sport as the primary predictor and the dimensions of EI as parallel mediators. The no-sport participant group served as the reference category for the multicategorical predictor, which included comparisons between individual vs. no-sport (X1) and team vs. no-sport (X2). Subsequent moderated-mediation models employed Model 59 in the PROCESS macro, testing sex (dichotomous) and age (continuous) as separate moderators of all model paths. The 10,000-sample percentile bootstrap procedure generated bias-corrected standard errors and 95% confidence intervals for all indirect effects, conditional indirect effects, and indices of moderated mediation. Indirect effects were considered significant when the 95% CI excluded zero (43).

## 3 Results

Table 1 presents the descriptive statistics, internal consistency coefficients, and bivariate correlations among the study variables.

**Table 1:** Descriptive statistics, macdonald's Omega, and bivariate correlations.

Variable	*M*	*SD*	ω	1	2	3	4	5	6	7
1. EI Self-emotion appraisal	15.53	2.46	0.74	—						
2. EI Others’ emotion appraisal	15.48	2.40	0.73	0.18**	—					
3. EI Use of emotion	15.35	2.77	0.80	0.45**	0.12*	—				
4. EI Regulation of emotion	13.47	3.17	0.80	0.37**	0.01	0.36**	—			
5. Fear of Negative Evaluation	18.52	4.94	0.88	−0.09**	0.08*	−0.16**	−0.07*	—		
6. Social Avoidance and Distress in New Situations	23.61	6.98	0.82	−0.10**	0.06*	−0.19**	−0.08*	0.63**	—	
7. Social Avoidance and Distress in General	9.09	3.62	0.83	−0.15**	−0.07*	−0.16**	−0.01	0.58**	0.64**	—
Sex (0 = girls, 1 = boys)	—	—	—	0.18**	−0.21*	0.17*	0.25**	−0.05	−0.12**	0.01
Age	—	—	—	−0.08*	0.10**	−0.09*	−0.02	−0.10**	−0.05	−0.06*

EI, emotional intelligence.

* *p* < 0.05.

** *p* < 0.01.

The dimensions of EI exhibited moderate and positive intercorrelations, except for the non-significant relationship between others' emotion appraisal and the regulation of emotion. Additionally, higher scores in self-emotion appraisal and the use of emotion were weakly to moderately associated with lower levels of social anxiety, whereas others' emotion appraisal and regulation of emotion displayed small to nonsignificant associations. Boys tended to report higher scores in self-focused dimensions of EI and lower levels of social avoidance and distress in new situations than their female counterparts. Age showed small and positive associations with others' emotion appraisal, while demonstrating small and negative associations with self-emotion appraisal, use of emotion, fear of negative evaluation, and social avoidance and distress in general situations.

Table 2 displays the direct and indirect effects of individual and team sport participation on the three social anxiety outcomes, mediated by the four dimensions of EI, including unstandardized path coefficients, standard errors, and bias-corrected confidence intervals.

**Table 2:** Path coefficients, standard errors, and confidence intervals for the mediation model.

Model paths	Fear of negative evaluation			Social avoidance and distress in new situations			Social avoidance and distress in general		
	*B*	*SE*	95% CI	*B*	*SE*	95% CI	*B*	*SE*	95% CI
*Direct effects*									
*a*_1_ (X1 → SEA)	0.11	0.22	−0.32 to 0.53	0.11	0.22	−0.32 to 0.53	0.11	0.22	−0.32 to 0.53
*a*_2_ (X1 → OEA)	0.06	0.21	−0.36 to 0.48	0.06	0.21	−0.36 to 0.48	0.06	0.21	−0.36 to 0.48
*a*_3_ (X1 → UOE)	0.77*	0.24	0.30 to 1.24	0.77*	0.24	0.30 to 1.24	0.77*	0.24	0.30 to 1.24
*a*_4_ (X1 → ROE)	−0.15	0.28	−0.70 to 0.40	−0.15	0.28	−0.70 to 0.40	−0.15	0.28	−0.70 to 0.40
*a*_5_ (X2 → SEA)	0.66*	0.20	0.27 to 1.05	0.66*	0.20	0.27 to 1.05	0.66*	0.20	0.27 to 1.05
*a*_6_ (X2 → OEA)	−0.33	0.20	−0.72 to 0.05	−0.33	0.20	−0.72 to 0.05	−0.33	0.20	−0.72 to 0.05
*a*_7_ (X2 → UOE)	1.40*	0.22	0.97 to 1.84	1.40*	0.22	0.97 to 1.84	1.40*	0.22	0.97 to 1.84
*a*_8_ (X2 → ROE)	0.62*	0.26	0.12 to 1.12	0.62*	0.26	0.12 to 1.12	0.62*	0.26	0.12 to 1.12
*b*_1_ (SEA → DV)	−0.13	0.10	−0.33 to 0.07	−0.07	0.07	−0.21 to 0.08	−0.17*	0.05	−0.27 to −0.06
*b*_2_ (OEA → DV)	0.33*	0.09	0.15 to 0.50	0.18*	0.06	0.05 to 0.30	−0.05	0.05	−0.14 to 0.04
*b*_3_ (UOE → DV)	−0.42*	0.09	−0.60 to −0.24	−0.31*	0.06	−0.43 to −0.18	−0.19*	0.05	−0.28 to −0.10
*b*_4_ (ROE → DV)	−0.01	0.07	−0.16 to 0.13	−0.01	0.05	−0.11 to 0.10	0.09*	0.04	0.01 to 0.17
*c*’_1_ (X1 → DV)	−0.54	0.61	−1.73 to 0.66	−0.69	0.43	−1.54 to 0.16	−0.25	0.32	−0.88 to 0.37
*c*’_2_ (X2 → DV)	1.63*	0.57	0.52 to 2.74	−0.40	0.40	−1.19 to 0.39	0.39	0.30	−0.19 to 0.97
*Indirect effects*									
*a*_1_*b*_1_ (X1 → SEA → DV)	−0.01	0.04	−0.12 to 0.06	−0.01	0.02	−0.06 to 0.04	−0.02	0.04	−0.11 to 0.06
*a*_1_*b*_2_ (X1 → OEA → DV)	0.02	0.07	−0.12 to 0.17	0.01	0.04	−0.07 to 0.10	−0.01	0.02	−0.04 to 0.03
*a*_1_*b*_3_ (X1 → UOE → DV)	−0.32*	0.14	−0.63 to −0.09	−0.24*	0.10	−0.45 to −0.07	−0.15*	0.06	−0.29 to −0.04
*a*_1_*b*_4_ (X1 → ROE → DV)	0.00	0.03	−0.06 to 0.06	0.00	0.02	−0.05 to 0.04	−0.01	0.03	−0.09 to 0.04
*a*_2_*b*_1_ (X2 → SEA → DV)	−0.09	0.08	−0.25 to 0.05	−0.04	0.05	−0.15 to 0.05	−0.11*	0.05	−0.21 to −0.03
*a*_2_*b*_2_ (X2 → OEA → DV)	−0.11	0.07	−0.26 to 0.01	−0.06	0.04	−0.16 to 0.01	0.02	0.02	−0.02 to 0.07
*a*_2_*b*_3_ (X2 → UOE → DV)	−0.59*	0.16	−0.92 to −0.29	−0.43*	0.11	−0.66 to −0.23	−0.27*	0.08	−0.44 to −0.12
*a*_2_*b*_4_ (X2 → ROE → DV)	−0.01	0.05	−0.12 to 0.10	−0.01	0.04	−0.09 to 0.08	0.06*	0.04	0.01 to 0.14

X1, individual sport vs. no-sport; X2, team sport vs. no-sport; SEA, self-emotion appraisal; OEA, others’ emotion appraisal; UOE, use of emotion; ROE, regulation of emotion; DV, Dependent/outcome variable.

* *p* < 0.05 (95% CI excluded zero).

The results of the mediation analyses for Model 1 (fear of negative evaluation), Model 2 (social avoidance and distress in new situations), and Model 3 (social avoidance and distress in general) indicated that EI dimensions, particularly the use of emotion, were key mechanisms linking sport participation to social anxiety outcomes. Across all three models, the indirect effects through the use of emotion were consistently negative and statistically significant for both individual and team sport participation (e.g., Model 1: individual sport, *B* = −0.32, 95% CI [−0.63, −0.09]; team sport, *B* = −0.59, 95% CI [−0.92, −0.29]), indicating that a greater use of emotion partially contributed to lower fear of negative evaluation and reduced social avoidance and distress. In Model 3, additional mediational pathways emerged for team sports: self-emotion appraisal significantly mediated the association between team sport participation and lower social avoidance and distress in general situations, whereas emotion regulation mediated a small but significant increase in the same outcome. On the other hand, individual sport participation showed no significant direct associations with any of the three social anxiety indicators once mediators were included. Conversely, team sport participation showed a substantial positive direct association with fear of negative evaluation (*c*'2 = 1.63, 95% CI [0.52, 2.74]) along with a significant negative indirect effect via use of emotion. This pattern of relationships, characterized by direct and indirect pathways operating in opposite directions, is indicative of inconsistent mediation. Specifically, participation in team sports is concurrently associated with a higher fear of negative evaluation via its direct pathway, while simultaneously related to a lower fear of negative evaluation through improved emotional processing.

The results of the moderated-mediation analysis indicated that sex showed no significant moderation of the direct or indirect effects of sport participation on social anxiety outcomes through the use of emotion (*p* > 0.05 for all interactions and indices of moderated mediation). However, some subgroup-specific conditional effects were observed. The direct relationship of team sport participation with higher fear of negative evaluation was only significant in boys (*conditional effect* = 1.69, 95% CI [0.25, 3.14]). Among girls, both individual and team sports were associated with lower fear of negative evaluation via the use of emotion, whereas team sports participation was also associated with lower social avoidance and distress in new and general situations (all 95% CI excluded zero). Among boys, both individual and team sports were linked to lower social avoidance and distress in new situations through the use of emotion (all 95% CI excluded zero).

Moderated-mediation analysis was also conducted to examine whether age moderated the direct and indirect effects of sport type on social anxiety dimensions. The conditional direct effects indicated that individual sport participation had a significant negative effect on fear of negative evaluation at age 16 only (*conditional effect* = −2.55, 95% CI [−4.61, −0.49]), whereas team sports showed significant positive (yet adverse) effects at ages 13–14 (*conditional effects* > 1.51, *p* < 0.02) that attenuated by age 16 (*p* = 0.23), suggesting a transient experience of social stress during early adolescence involvement in team sports. The conditional indirect effects, mediated by the use of emotion, were consistently negative and significant for team sports across all social anxiety outcomes and age groups (*conditional effects* < −0.25, with all 95% CI excluding zero), whereas for individual sports, these indirect effects were only significant at ages 13–14 (*conditional effects* < −0.13, with all 95% CI excluding zero). Collectively, these findings indicate that while the associations of team sport participation with social anxiety are age-specific and occasionally adverse among younger adolescents, the indirect protective mechanism through the use of emotion operates consistently throughout adolescent development, mitigating social anxiety regardless of age.

## 4 Discussion

The primary aim of the present study was to examine whether dimensions of EI serve as mediators in the relationships between sport type and social anxiety among adolescents, and whether these indirect and direct pathways are influenced by sex or age. Overall, the findings provide novel empirical support for a conditional process model, in which the use of emotion emerged as the primary psychological mechanism linking sports participation to reduced social anxiety levels. Age further moderates this relationship in both direct and indirect pathways.

The most consistent finding across all three social anxiety outcomes was the significant negative indirect effect of both individual and team sport participation via the use of emotion. These results align with previous evidence suggesting that EI plays a crucial mediating role in reducing social anxiety by mitigating the intensity of negative emotions and improving interpersonal functioning (31–33). Moreover, this finding builds on our previous research with the same sample (23), which showed that the effects of youth sports participation on positive mental health indicators (i.e., self-esteem and life satisfaction) were fully mediated by the EI dimensions of use of emotion and self-emotion appraisal. The use of emotion dimension represents the self-motivational component of EI, reflecting the individuals' ability to effectively harness their emotions to facilitate cognitive processes and improve behavioral performance (46). As such, these self-regulatory mechanisms contribute to more adaptive appraisals of socially threatening situations and a lower likelihood of engaging in maladaptive strategies such as expressive suppression and rumination (9, 34, 35). Notably, the indirect effects for team sports were approximately twice the magnitude of those for individual sports (e.g., fear of negative evaluation: *B* = −0.59 vs. −0.32), suggesting that the collective and interdependent nature of team sport environments may be particularly effective in promoting this emotional capacity and its associated outcomes (23, 28, 30). This finding is consistent with previous research indicating that team sports provide wider opportunities for emotional co-regulation, social perspective-taking, sustained collaborative effort, and conflict resolution (17–19, 29).

A second key contribution of this study is the identification of inconsistent mediation regarding the effects of team sport on fear of negative evaluation. In contrast to individual sport participation, which showed no significant direct associations with any dimension of social anxiety, team sport participation demonstrated a substantial positive (yet detrimental) direct effect on fear of negative evaluation, accompanied by a significant negative indirect effect mediated by the use of emotion. This duality, in which direct and indirect effects operate in opposite directions, exemplifies inconsistent (or competitive) mediation (43) and suggests that team sports can simultaneously increase and alleviate the fears of negative evaluations through distinct processes and sociocontextual influences. On the one hand, team sports may intensify the perception of public scrutiny and performance pressure, thereby increasing social-evaluative concerns, particularly in more competitive or performance-oriented environments (17, 20). Another perspective is that this pattern may partly reflect selection effects rather than the influence of the sport context itself. Adolescents with greater pre-existing sensitivity to social evaluation, or those whose participation is encouraged by families and peers who perceive team sports as socially beneficial, may be more likely to engage in these settings. On the other hand, team sports settings may also be associated with structured and repeated opportunities to use and develop emotional skills that are negatively linked to social anxiety (19, 28, 29). This observed concurrent effect may also help clarify previous inconsistent findings, including studies that reported no significant differences in social anxiety between team and individual sport participants (21, 22). Taken together, this evidence suggests that while team sports may offer socioemotional benefits for many adolescents, they can also be related to increased fears of negative evaluation, particularly among certain groups, such as males, younger individuals, or those with limited sports experience (38, 46). Nevertheless, further longitudinal research is needed to clarify these possibilities and to disentangle selection effects from the contextual influence of team sport participation.

The second aim of this study was to investigate whether sex moderated the direct and indirect pathways between sport type, EI, and social anxiety, which was not confirmed by the present findings. This evidence is consistent with a previous study (42), which reported that the protective associations between sport participation and social anxiety symptoms did not vary by sex. However, it contrasts with a larger body of literature suggesting that sex serves as a significant moderator in the development of both EI and social anxiety during adolescence (36, 37). In this regard, different explanations have been provided for the mediator effect of sex on emotions and anxiety, combining biological differences (e.g., brain activation) and cultural conditioning (e.g., gender role socialization), among others (49, 50). Nonetheless, in the present study, subgroup-specific conditional effects were observed at the level of specific pathways, and while these should be interpreted with caution due to the non-significant interaction tests, they provide theoretically meaningful insights. The adverse direct effect of team sports on the fear of negative evaluation was significant only for boys, while for girls, both types of sport participation were linked to a reduced fear of negative evaluation through the use of emotion dimension. These patterns may likely reflect the influence of gendered socialization norms, selection pressures, or perception of more public performance scrutiny, particularly prominent in male-dominated competitive sports (20, 38), making boys in team sports more susceptible to negative assessments from others in social situations. Regarding age-conditional effects, the results provide significant developmental insights into the relationship between sport type and social anxiety during adolescence. In early adolescence, team sport participation was associated with a transient increase in the direct pathway to fear of negative evaluation, a finding that likely reflects the heightened sensitivity to evaluation by significant others, along with increased self-consciousness and social comparison typical of this developmental stage (2, 6). It may also indicate that involvement in team sports does not necessarily facilitate sensitization to the social-evaluation aspects central to social anxiety in early adolescents (48). Simultaneously, the stable indirect protective mechanism associated with the use of emotion suggests that team sport environments may contribute to the gradual development of emotional skills, which may have the potential to provide a buffer against these social-evaluative pressures throughout adolescence (19, 28–30). Conversely, the protective direct effect of individual sports on fear of negative evaluation was observed exclusively in late adolescence, which may be attributed to the development of self-efficacy, self-esteem, and emotional regulation skills during this life period, suggesting that the context of individual sports helps adolescents in building resilience against social anxiety once certain emotional abilities are sufficiently developed (37, 39, 48). Moreover, the observed conditional indirect effects reinforce this idea, as the strategic use of emotions to enhance performance served as a mediator only during the early phase of adolescence.

The present study has some limitations that should be recognized, particularly its cross-sectional design, which restricts the ability to draw causal inferences. Given the exploratory nature of this research, relevant confounding factors such as years of sports experience, weekly practice frequency and intensity, competitive level, socioeconomic background, biological maturity, family support, and psychological well-being were not assessed and may influence the observed associations. Therefore, future studies should aim to replicate these findings across a broader range of populations and settings, including schools, communities, and informal sports.

In conclusion, the findings of the present study suggest that EI, particularly in relation to the use of emotion, may represent an important explanatory pathway linking sport participation and lower social anxiety in adolescents. Furthermore, engagement in both individual and team sports was shown to be indirectly associated with reduced fear of negative evaluation, as well as lower levels of social avoidance and distress, mediated through the use of emotion. Notably, while sex did not significantly influence the model's direct or indirect pathways, age revealed meaningful conditional effects. Team sport participation displayed transient adverse effects on fear of negative evaluation during early adolescence, whereas the protective indirect effects through EI remained consistent across different developmental stages. These findings underscore the significance of sport participation as a crucial context for the development of socio-emotional skills during adolescence, although some of these benefits seem to be influenced by contextual and developmental factors.

## Data Availability

The raw data supporting the conclusions of this article will be made available by the authors, without undue reservation.
